# Evaluation of 3D blood flow patterns and wall shear stress in the normal and dilated thoracic aorta using flow-sensitive 4D CMR

**DOI:** 10.1186/1532-429X-14-84

**Published:** 2012-12-13

**Authors:** Jonas Bürk, Philipp Blanke, Zoran Stankovic, Alex Barker, Maximilian Russe, Julia Geiger, Alex Frydrychowicz, Mathias Langer, Michael Markl

**Affiliations:** 1 Department of Diagnostic Radiology, Medical Physics, University Hospital, Freiburg, Germany; 2 Clinic of Radiology and Nuclear Medicine, University Hospital of Schleswig-Holstein, Campus, Lübeck, Germany; 3 Departments of Radiology and Biomedical Engineering, Northwestern University, Chicago, IL, USA

**Keywords:** 4D flow, Flow quantification, Aneurysm of the ascending aorta

## Abstract

**Background:**

The purpose of this study was to investigate 3D flow patterns and vessel wall parameters in patients with dilated ascending aorta, age-matched subjects, and healthy volunteers.

**Methods:**

Thoracic time-resolved 3D phase contrast CMR with 3-directional velocity encoding was applied to 33 patients with dilated ascending aorta (diameter ≥40 mm, age=60±16 years), 15 age-matched normal controls (diameter ≤37 mm, age=68±7.5 years) and 15 young healthy volunteers (diameter ≤30 mm, age=23±2 years). 3D blood flow was visualized and flow patterns were graded regarding presence of supra-physiologic-helix and vortex flow using a semi-quantitative 3-point grading scale. Blood flow velocities, regional wall shear stress (WSS), and oscillatory shear index (OSI) were quantified.

**Results:**

Incidence and strength of supra-physiologic-helix and vortex flow in the ascending aorta (AAo) was significantly higher in patients with dilated AAo (16/33 and 31/33, grade 0.9±1.0 and 1.5±0.6) than in controls (2/15 and 7/15, grade 0.2 ± 0.6 and 0.6 ± 0.7, *P*<.05) or healthy volunteers (1/15 and 0/15, grade 0.1 ± 0.3 *P*<.05). Greater strength of the ascending aortic helix and vortex flow were associated with significant differences in AAo diameters (*P*<.05). Peak systolic WSS in the ascending aorta and aortic arch was significantly lower in patients with dilated AAo (*P*<.0157-.0488). AAo diameter positively correlated to time to peak systolic velocities (r=0.30-0.53, *P*<.04), OSI (r=0.33-0.49, *P*<0.02) and inversely correlated to peak systolic WSS (r=0.32-0.40, *P*<.03). Peak systolic WSS was significantly lower in AAo aneurysms at the right and outer curvature within the AAo and proximal arch (*P*<.01-.05).

**Conclusions:**

Increase in AAo diameter is significantly correlated with the presence and strength of supra-physiologic-helix and vortex formation in the AAo, as well with decrease in systolic WSS and increase in OSI.

## Background

Thoracic aortic aneurysmal disease is frequent, frequently involving the aortic root and ascending aorta [1,2]. It can be complicated by dissection or rupture with an increased lifetime risk when the aortic diameters exceed 6 cm [1]. The mean growth rate of thoracic aneurysms is only about 0.1 cm/year, although there is substantial variation in individual aneurysm progression [3]. Established risk factors for an accelerated aneurysm growth rate include simple geometric markers such as initial size or localization. In addition, the presence of aortic valve disease (stenosis), congenital abnormalities (bicuspid aortic valve), or connective tissue disorders can influence aneurismal growth. However, predicting aneurysm progression is nearly impossible and dissection and rupture also occur at diameters under 6 cm [3,4].

Recent studies have focused on establishing alternative criteria for evaluating aneurysms, i.e. RNA expression patterns, biomarkers, aortic curvature geometry and mechanical properties of the aorta [1,5,6]. In this context, the assessment of aortic hemodynamics and the presence of altered flow patterns, as well as distribution and changes in wall shear stress (WSS) and the oscillatory shear index (OSI) and their association with changes in aorta size may provide further insights in how aneurysms develop and in assessing the risk of dissection [7,8]. These factors may play an important role in aneurysm development and can be assessed by time-resolved 3D phase contrast cardiovascular magnetic resonance (CMR) with 3-directional velocity encoding (flow-sensitive 4D CMR), also providing comprehensive information on aortic hemodynamics with 3D visualization of blood flow pattern [9-11].

The purpose of this study was to investigate 3D flow patterns and vessel wall parameters in the ascending aorta and aortic arch in correlation to the vessel geometry in patients with dilated ascending aorta, age-matched subjects, and healthy volunteers.

## Methods

### Study population

This study was institutional review board-approved; written informed consent was obtained from all participants. The study population consisted of three cohorts: 33 patients with dilated ascending aorta or AAo aneurysm (diameter >40 mm, 30 male, 3 female, mean age 59.8±15.9 years), 15 age-matched normal controls (AAo diameter <40 mm, 12 male, 3 female, mean age 67.1±7.5 years) and 15 healthy young volunteers (12 male, 3 female, mean age 22.7±1.6 years) were included. Patients and age matched controls were retrospectively included from a study cohort comprising 150 patients, examined between 2006 and 2009 for investigation of aortic blood flow patterns in stroke patients. 29% of the patients with dilated ascending aorta had transient ischemic attacks, 71% ischemic infarctions vs. 26%/74% of the age matched controls. Inclusion criteria were age 18 to 85 years and suitability for CMR examination at 3 Tesla. In our study we chose to include only aneurysm patients who did not have other cardiovascular abnormalities to focus on the relationship of hemodynamics and aortic dilatation. Exclusion criteria’s were ejection fraction (EF) <35%, aortic valve disease, determined by transesophageal echocardiography or known connective tissue disorders. The study cohort, as well as inclusion and exclusion criteria, was also previously described [12-14].

### CMR

**Figure 1:**
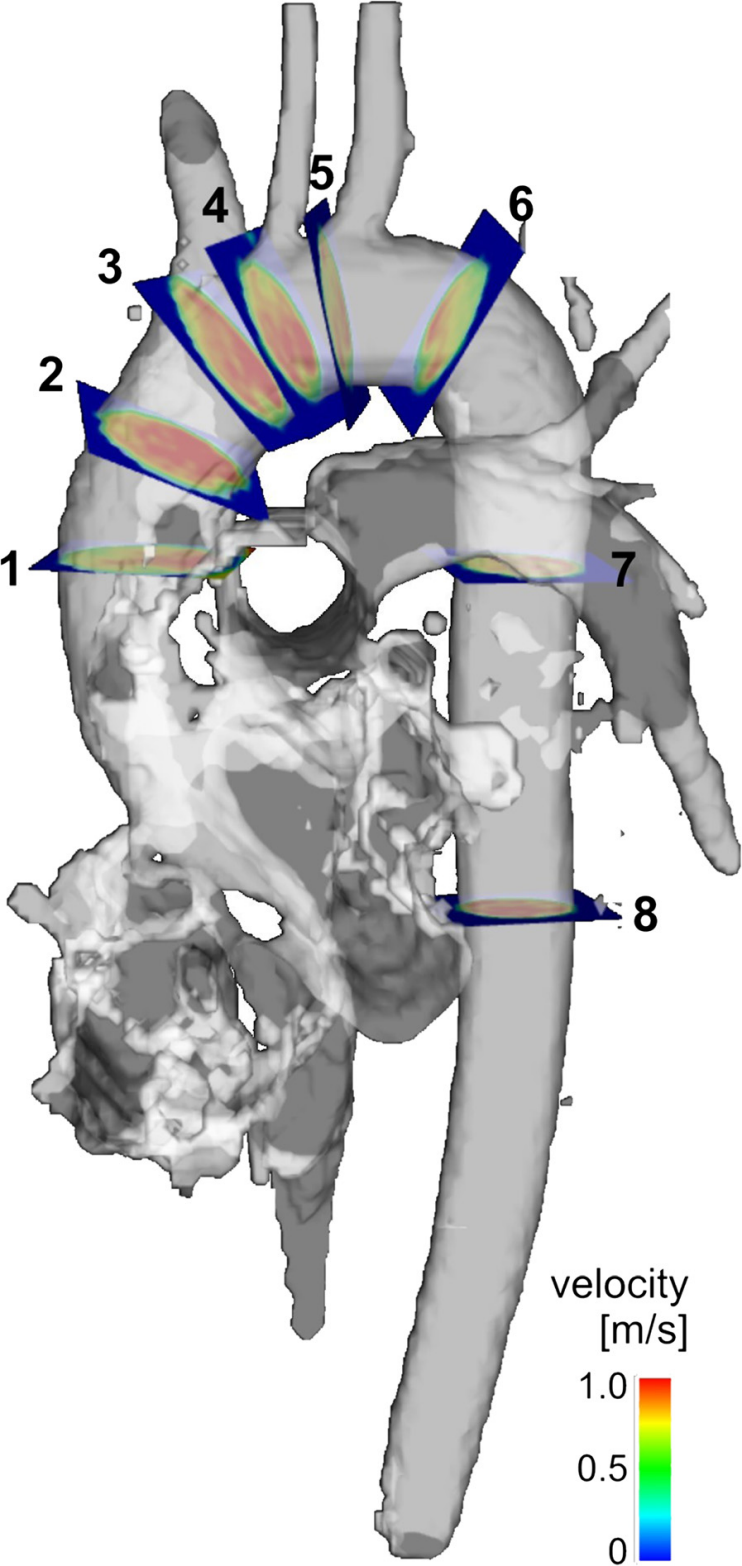
Position of the planes for particle trace visualization (1, 3 and 6) and blood flow/ vessel wall quantification (1–8) in the thoracic aorta.

All examinations were performed on a 3 T MR System (Siemens, Erlangen, Germany) using flow-sensitive 4D CMR based on an RF-spoiled gradient-echo sequence with interleaved three-directional velocity encoding. All measurements were prospectively gated to the ECG. Scanning was performed during free breathing using navigator respiratory gating based on diaphragm motion as described previously [15]. Data were acquired in a sagittal oblique 3D slab encompassing the thoracic aorta. Imaging parameters were as follows: venc =150 m/s, spatial resolution =1.6-2.2 × 2.1-3.2 × 2.4-3.5 mm^3^, field of view 400 × 300 mm^2^, flip angle α=7-15°, echo time (TE) =2.6-3.5 ms, repetition time (TR) =5.1-6.1 ms, temporal resolution =40.8-48.8 ms, axial-oblique 3D Volume. Diameters of the ascending aorta (AAo), arch, and proximal descending aorta (DAo) were determined by electronic caliper measurements from additional morphological CMR data, using a T1-weighted radiofrequency-spoiled and fat-saturated 3D gradient echo sequence with an isotropic resolution of 1 mm^3^. Diameters were measured at the level of analysis planes number 1,5 and 8 (see Figure 1). In order to minimize wall motion and breathing artifacts, data were acquired during a short diastolic window (157–158 ms) combined with respiratory navigator gating. Imaging parameters were as follows: voxel size = 0.8 × 1.1 × 1.1 mm3; field of view = 350 × 252 mm^2^; echo time/pulse repetition time = 1.7-2.3/5.1-5.5 ms; flip angle α=20°.

### Data processing and 3D blood flow visualization

**Figure 2:**
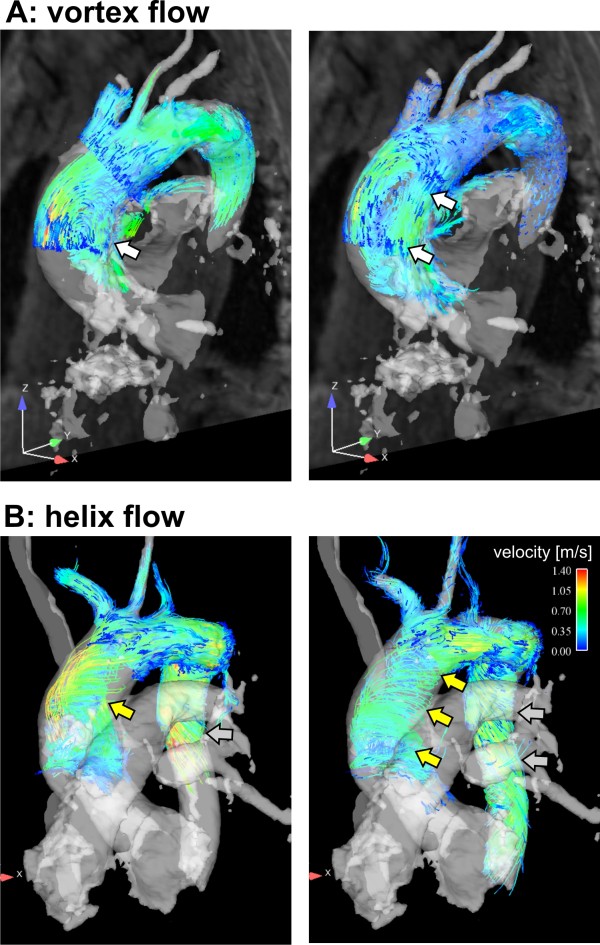
**Vortex and supra-physiologic-helix flow.** Early (left column) and late (right column) systolic time-resolved particle traces show the formation of a large flow vortex encompassing the entire AAo (**A**, white arrows, grading = 1.5) in an aneurysm patient (AAo diameter = 41 mm). **B**: Development of supra-physiologic-helix flow (yellow arrows, grading=2) in the ascending (diameter=48 mm) and descending aorta in another aneurysm patient (gray arrows).

Data processing included eddy current and velocity aliasing correction and the calculation of a 3D phase contrast angiogram (3D PC-MRA) from the 4D data (Figure 1, gray-shaded iso-surfaces) [16]. Flow pattern analysis (EnSight, CEI, Apex, USA) was based on time-resolved particle traces originating from a total of three predefined, manually positioned emitter planes, positioned at the following locations as outlined in Figure 1: in the proximal ascending aorta at the level of the pulmonary artery (Plane 1), in the ascending aorta proximal to the origin of the brachiocephalic trunk (Plane 3), and in the proximal descending aorta (Plane 6). Particle traces visualize the path of virtual massless particles released from the emitter planes in spatially and temporally varying, three-directional blood flow velocities. The resulting traces resembled the temporal and spatial evolution of 3D blood flow in the aortic lumen. Vortex flow was defined as revolving particles around an axis in orthogonal to the vessel centreline (Figure 2A). Helix flow was considered a rotational motion around the longitudinal axis of the vessel centreline (i.e. the physiologic flow direction) creating a corkscrew-like flow pattern (Figure 2B).

Visual grading was performed by rotating the particle traces in 3D and by investigating the rotating flow by viewing along the axis or helix or vortex flow in the thoracic aorta in 3 segments: a) the ascending aorta, 2) the arch region, and 3) the descending aorta. A number of studies have shown that left ventricular outflow results in right handed helical flow in the ascending aorta during ventricular systole, as a physiological phenomenon [17,18]. However, normal aortic helix flow can be considered moderate, with a flow rotation typically <180°. Also vortex flow can occur rarely as a physiological flow phenomenon in the AAo [7]. Helix and vortex severity were graded in a blinded fashion by two independent observers using a three point grading-scale: 0, small helix/vortex formation (flow rotation <180°); 1, moderate supra-physiologic-helix/vortex (flow rotation <360°); 2, pronounced supra-physiologic-helix/vortex (flow rotation >360°). For helix flow, the direction of flow rotation (right- or left-handed, when viewed orthogonally to the vessel axis in the direction of blood flow) was noted. For each detected vortex flow pattern it was noted whether the vortex filled the aortic lumen partly or fully.

### Flow velocity and wall shear stress quantification

**Figure 3:**
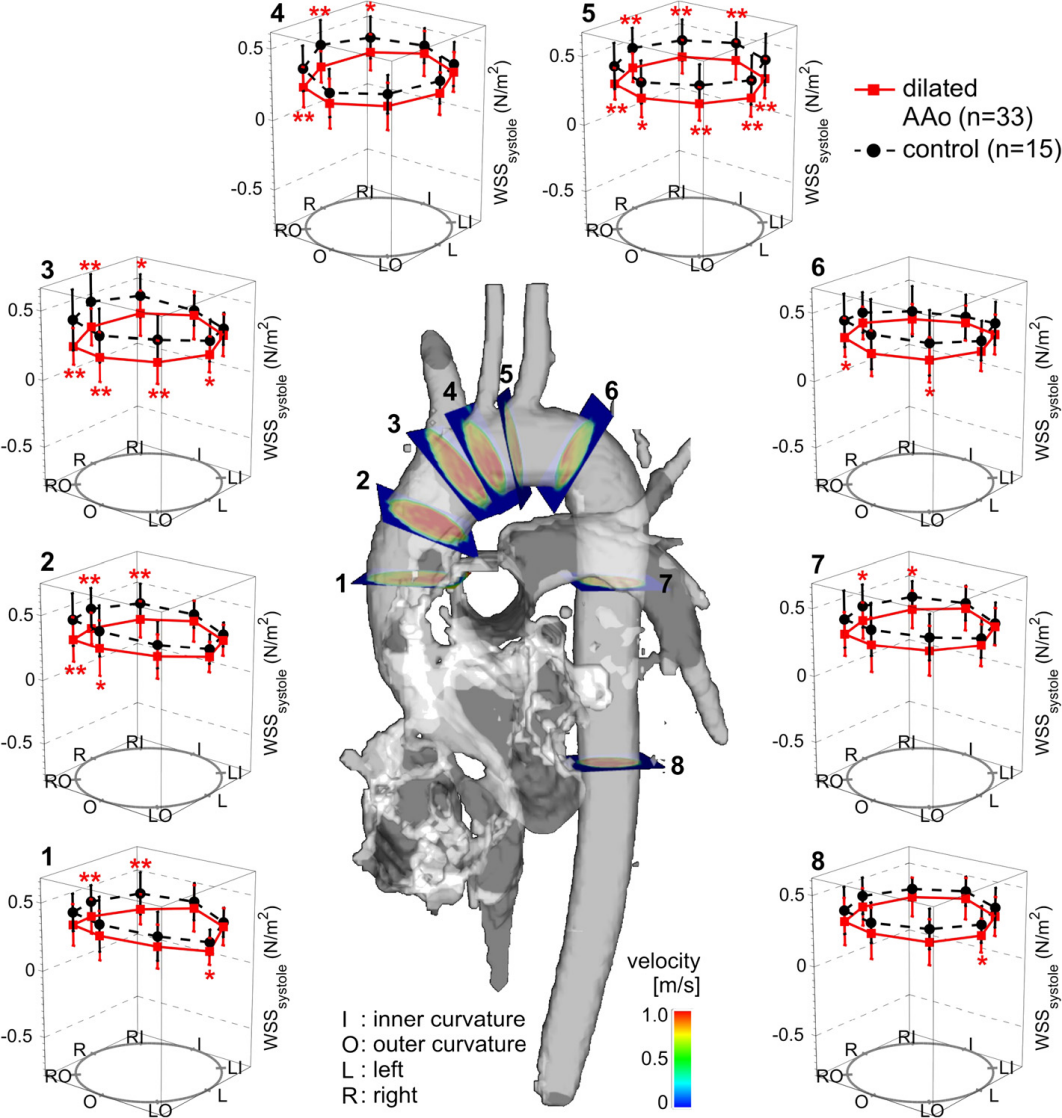
**Distribution of regional peak systolic wall shear stress (WSS) in the ascending aorta (AAo) in aneurysm patients (n=33) compared to age-matched controls (n=15).** I=inner curvature, O=outer curvature, L=left, R=right. */** indicate significant differences with *p*<.05 / *p*<.01.

We used the reconstructed 3D PC-MRA to manually position eight analysis planes at defined anatomical landmarks in the thoracic aorta at the following positions as illustrated in Figure 1. Plane numbers 1 and 3: at the aforementioned locations. Plane 2: on the half way between plane 1 and 3. Plane 4: between the origins of the brachiocephalic trunk and left common carotid artery. Plane 5: between the origins of the left common carotid and left subclavian arteries. Plane 6: in the proximal descending aorta. Plane 7: at the level of plane 1 and plane 8 in the distal descending aorta. Plane 1, 3 and 6 were simultaneously used for flow visualization and quantification. The aortic lumen contours were manually delineated for each analysis plane, on all time-frames using a home-built tool programmed in Matlab (Matlab, The Mathworks, USA). We calculated velocity-time curves, peak systolic velocity (cm/sec), time-to-peak (TTP) systolic velocity (msec), and retrograde flow (ml/min). Retrograde flow was quantified at the location of the analysis planes. All analyses planes were angulated and positioned perpendicular to the local vessel axis. The wall shear stress (WSS) estimate was based on a direct interpolation of the local velocity derivative on the segmented vessel lumen contour using b-splines as described previously [19]. Based on a consistent orientation of the analysis planes, regional time-resolved WSS vectors were mapped onto a standardized 8-quadrant model representing 8 angular segments of the aortic wall: outer and inner curvature, right outer and left outer curvatures, right inner and left inner curvatures and right and left (Figure 3). In addition the time-averaged WSS magnitude and peak systolic WSS_systole_ magnitude for each analysis plane and each segment, and the oscillatory shear index (OSI), reflecting the degree of WSS inversion over the cardiac cycle, as previously defined by He and Ku [20], were calculated.

### Statistical analysis

All statistical analyses were performed using commercially available software (SPSS 17.0; SPSS Inc., Chicago, Ill). Kolmogorov-Smirnov tests were performed to test for equal distribution of continuous variables. Continuous variables were expressed as mean ± standard deviation. Categorical variables were expressed as frequencies and percentages. To detect statistically significant differences between the distributions of the grading between patients and control groups, the Mann–Whitney U test was employed. Continuous variable were compared using an unpaired two-sided *t*-test. Differences with *P*<.05 were considered significant. The relationship between ascending aortic diameter and hemodynamics parameters was investigated using correlation analysis based on linear regression. The regression’s overall quality was assessed using Pearson’s correlation coefficient r. A p value <.05 was considered statistically significant. Interobserver agreement on semi-quantitative grades of helix or vortex formation between the two readers was calculated using κ statistics. A κ value over 0.81 corresponded to excellent interobserver agreement, with values of 0.61–0.80 corresponding to good agreement [21].

## Results

There were no significant differences in mean age (median age 62±15.9 vs. 67±7.5 *P*=.0966), body mass index (median BMI 26.4±4.2 vs. 26.2±3.4 *P*=.927), EF (median EF 55±6.6 vs. 60±4.2 *P*=.155) and heart rate (median heart rate 71±8.8 vs. 65±5.7 *P*=.161) in the dilated AAo or age-matched controls. The majority of patients with dilated AAo and age matched controls were hypertensive and received blood pressure medication (86% vs.93%). 53% of the patients with dilated AAo and 46% of the age matched controls were hyperlipidemic. The mean AAo diameter as well as the AAo/DAo diameter ratio was significantly higher in the dilated AAo than in age-matched controls (*P<*.01) and healthy young volunteers (*P*<.01). As expected due to the physiological growth of the aorta with time [22], diameters of the AAo, aortic arch and DAo were significantly higher in the age-matched controls than in the healthy volunteers (*P<*.01), whereas the AAo/DAo diameter ratio was not significantly different (*P*=.9801, Table 1).

**Table 1:** Aortic geometry in aneurysm patients and control groups

	**Patients**	**Age-matched**	**Healthy**	***t*-test [*p*-value]**		
**Aortic geometry**	**dilated aorta**	**controls**	**volunteers**	**1 vs. 2**	**1 vs. 3**	**2 vs. 3**
**AAo [mm]**	44 ± 4*^#^	35 ± 2^+^	27 ± 2	<.01	<.01	<.01
	(40–51)	(32–37)	(23–30)			
**arch [mm]**	30 ± 4*^#^	28 ± 3^+^	22 ± 2	.04	<.01	<.01
	(24–43)	(25–35)	(18–24)			
**DAo [mm]**	28 ± 4^#^	27 ± 2^+^	21 ± 2	.39	<.01	<.01
	(19–41)	(25–31)	(17–24)			
**AAo / DAo ratio**	1.6 ± 0.3*^#^	1.3 ± 0.1	1.3 ± 0.2	<.01	<.01	.98
	(1.1 - 2.7)	(1.2 - 1.4)	(1.0 - 1.8)			

**Note. -** Diameters of the AAo and AAo/DAo diameter ratio of the study cohorts.

*: significant differences (*p*<.05) between aneurysm patients and age matched controls.

**#**: significant differences (*p*<.05) between aneurysm patients and healthy volunteers.

+: significant differences (*p*<.05) between age-matched controls and healthy volunteers.

### Visual grading of blood flow

**Figure 4:**
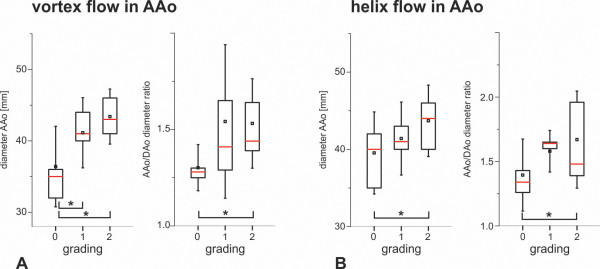
**Box plot of the ascending aorta diameter and AAo/Dao diameter ratio in relation to the subjective vortex and helix flow grading for AAo aneurysm patients (n=48).** Box = first to third quartiles. Red line = median. Whiskers = minimum and maximum values. * indicates significant differences (*P* <.05) by means of student t test.

Particle trace visualization was successfully performed in all volunteers. Inter-observer variability for flow pattern grading was excellent (κ=0.86). Supra-physiologic-helix formation in the ascending aorta was significantly more prevalent in patients with dilated AAo than in age-matched controls and young healthy volunteers (*P*=.004 and *P*=.003, Table 2). Vortex flow was noted in the ascending aorta in all but one of the patients with dilated AAo (32/33, 94%) but in less than half of the age-matched controls (7/15, 47%) and not at all in the young healthy volunteers (Figure 2, Table 2). Vortex strength was significantly higher in patients with dilated AAo than in age-matched controls (average grading of 1.5±0.6 vs. 0.6±0.7, p=.004). In all cohorts, supra-physiologic-helix and vortex flow was observed less frequently in the aortic arch and descending aorta than in the ascending aorta (Table 2). When present, helix flow was right-handed in all but one patient. In patients with dilated AAo, supra-physiologic-helix and vortex flow was observed simultaneously in 15/33 (45%) in the AAo and in 2/33 (6%) in the aortic arch. Simultaneous occurrence of vortex and supra-physiologic-helix flow was not seen in age-matched controls or healthy volunteer. Simultaneous occurrence was not present in the DAo in all three cohorts. In both, patients with dilated AAo and age-matched controls presence and strength of the ascending aortic supra-physiologic-helix and vortex formation was directly associated with an increase in AAo diameter and AAo/DAo diameter ratio (Figure 4).

**Table 2:** Incidence of supra-physiologic-helix and vortex pattern

**incidence [n (%)]**	**Helix**			**Vortex flow**		
**grading**	**AAo**	**arch**	**DAo**	**AAo**	**arch**	**DAo**
**patients, dilated aorta**	16 (48%)*^#^	5 (15%)*^#^	3 (9%)	31 (94%)*^#^	4 (12%)	3 (9%)
**n=33**						
**age-matched controls**	2 (13%)	2 (13%)	2 (13%)	**7 (47%)**^**+**^	2 (13%)	1 (7%)
**n = 15**						
**healthy volunteers**	1 (7%)	none	none	none	none	none
**n = 15**						

**Note. -** Table 2 shows the incidence of helix and vortex flow in the AAo (ascending aorta), aortic arch and DAo (descending aorta) of the three study cohorts.

*: significant differences (*p*<.05) between aneurysm patients and age-matched controls.

**#**: significant differences (*p*<.05) between aneurysm patients and healthy volunteers.

+: significant differences (*p*<.05) between age matched controls and healthy volunteers.

### Quantifying flow velocity and wall shear stress

**Figure 5:**
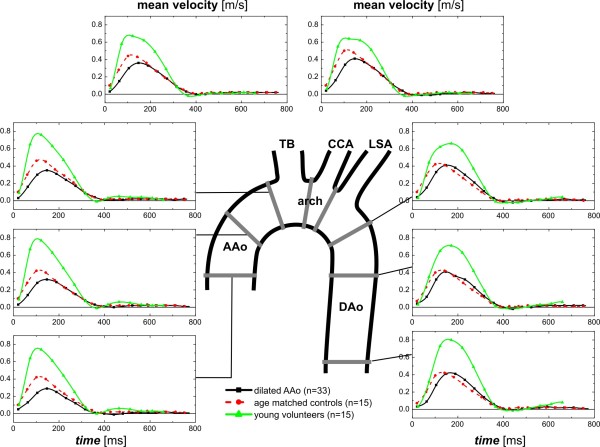
**Dynamics of mean blood flow velocities in eight standardized analysis planes distributed along the thoracic aorta.** The central illustration defines the position of the analysis planes. Graphs depict blood flow at each location over the cardiac cycle averaged for healthy volunteers (green curves), AAo patients (black curves) and aged-matched controls (red curves) TB=brachiocephalic trunk, CCA=common carotid artery, LSA=left subclavian artery.

**Figure 6:**
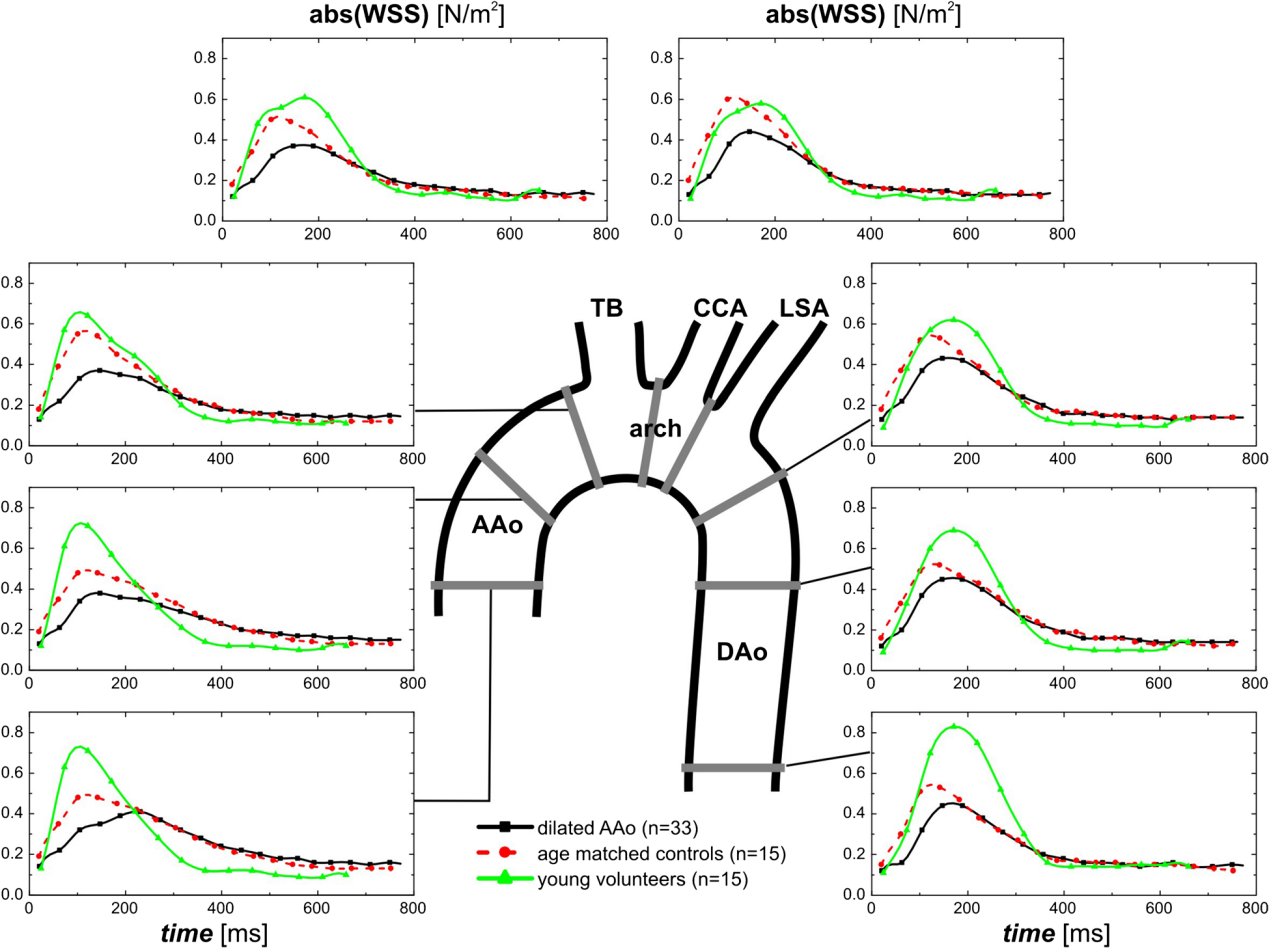
**Temporal evolution of absolute wall shear stress (abs. WSS) averaged over the lumen circumference for each analysis plane in eight standardized analysis planes distributed along the thoracic aorta as depicted by the central illustration.** Graphs depict WSS at each location over the cardiac cycle averaged for healthy volunteers (green curves), AAo patients (black curves) and aged matched controls (red curves). TB=brachiocephalic trunk, CCA=common carotid artery, LSA=left subclavian artery.

Pulsatile mean velocity-time curves and dynamics of WSS magnitude over the cardiac cycle are depicted in Figures 5 and 6 for the eight analysis planes in the thoracic aorta. For both blood flow velocity and WSS, peak systolic values were reduced and delayed in patients and age matched controls, but not in young healthy volunteers.

**Figure 7:**
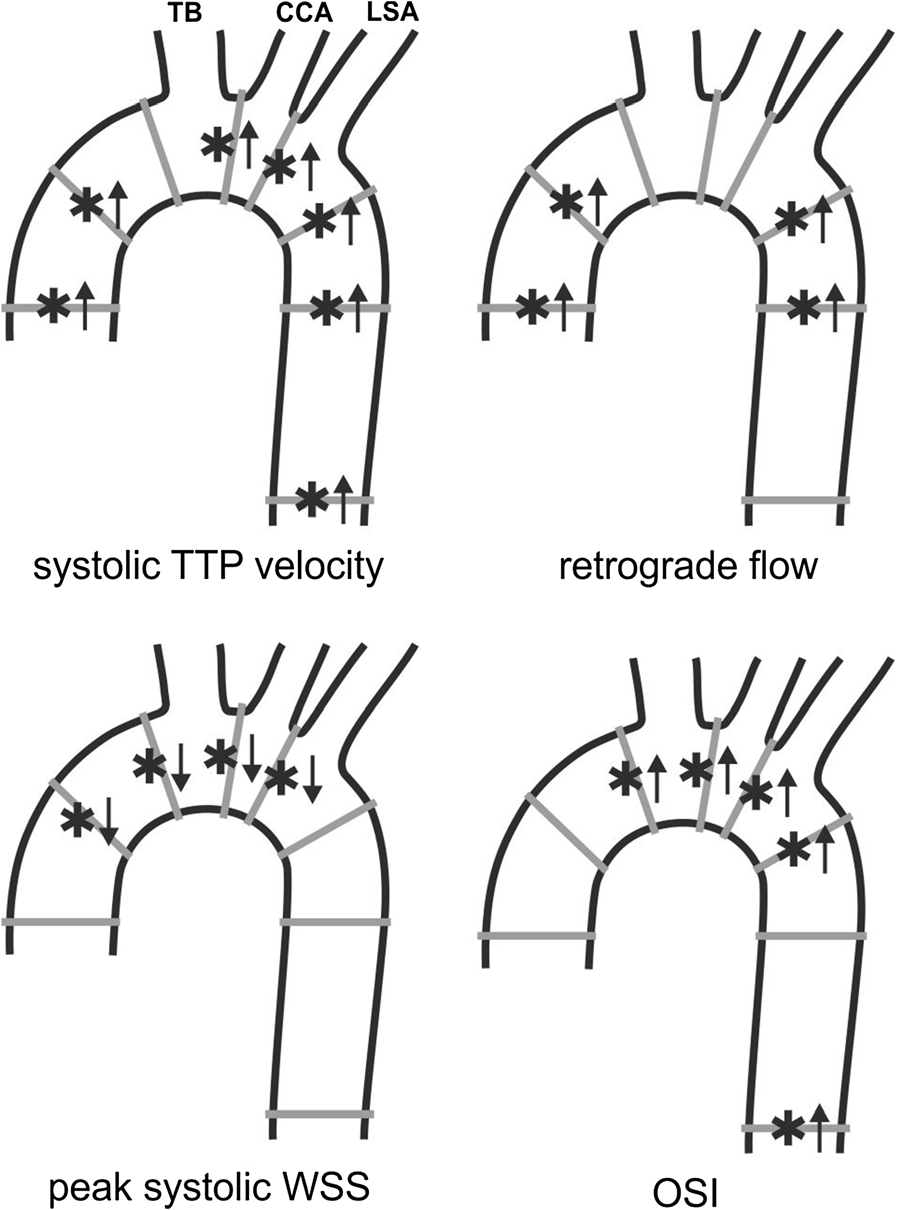
**Regional differences in flow and wall parameters in patients with dilated ascending aorta (AAo) compared to age-matched controls.** Arrows (*↑/*↓) indicate significant increase/ decrease (*P*<.05). TB=brachiocephalic trunk, CCA=common carotid artery, LSA=left subclavian artery.

Moreover, patients with dilated AAo and age-matched controls demonstrated several significant differences in regional flow velocities and wall parameters as summarized in Figure 7 and Table 3. In aneurysm patients, systolic time to peak (TTP) velocity was significantly increased in 7 analyzing planes (*P*=.0044-.0321) and retrograde flow was significantly increased in four analyzing planes, when compared to age-matches controls. In addition, peak systolic WSS was significantly decreased in the ascending aorta and entire aortic arch in aneurysm patients compared to the two other cohorts. Conversely, OSI was significantly higher in analogous locations in the thoracic aorta (Figure 7). There was no significant difference in time-averaged WSS magnitude between patients and age-matched controls in most of the analysis planes except for plane 5 in the aortic arch. These findings imply a systematic and significant reduction in WSS and an increase in OSI in patients with dilated AAo.

**Table 3:** Differences in regional flow velocities and vessel wall parameters

**Plane**	**p-values patients vs. age-matched controls**			
	**Systolic TTP**	**Retrograde flow**	**Peak systolic**	**OSI**
	**Velocity (msec)**	**(ml/min)**	**WSS (N/m**^**2**^**)**	**(a.u.)**
**1**	**.0044**	**.0367**	.1623	.6837
**2**	**.0173**	**.0487**	**.0488**	.1190
**3**	.0541	.0850	**.0384**	**.0341**
**4**	**.0123**	.0845	**.0337**	**.0430**
**5**	**.0126**	.0677	**.0157**	**.0070**
**6**	**.0086**	**.0266**	.2128	**.0184**
**7**	**.0322**	**.0301**	.2623	.1101
**8**	**.0221**	.1194	.0717	**.0187**

**Note. -** Table 3 shows differences in regional flow velocities and vessel wall parameters between aneurysm patients and age-matched controls in all of the 8 analysis planes. Significant differences (*P* <.05) in bold print.

Correlation analysis revealed a weak to moderate yet significant correlation between the degree of aortic dilation [cm] and prolonged TTP systolic velocities in all eight analysis planes (r=0.30-0.53, *P*<.04). Similarly, OSI correlated significantly with AAo diameter in all analysis planes except for the plane in the proximal AAo (r=0.33-0.49, *P*<.02). In addition, peak systolic WSS demonstrated a weak but significant inverse correlation with increased AAo diameters in four analysis planes in the ascending aorta and aortic arch (r=0.32-0.40, *P*<.03).

To detect segmental differences over the lumen circumference, the distribution of wall parameters of each analysis plane of the dilated AAo group and the age-matched controls was evaluated. Figure 3 shows the segmental distribution of peak systolic WSS in the entire thoracic aorta in patients with dilated AAo compared to age-matched controls. A significant decrease in regional peak systolic WSS in patients - pronounced in the right-outer curvature in the AAo and proximal arch (planes 1–4) was observed. Differences were less pronounced in the descending aorta (planes 6–8).

## Discussion

In this study, flow-sensitive 4D CMR was used to visualize different flow patterns in the thoracic aorta of 33 patients with dilated ascending aorta (≥40 mm), 15 age-matched normal controls, and 15 young healthy volunteers. Our aim was to provide insight into alterations in blood flow and vessel wall parameters in the dilated aorta compared to young and age-matched controls. In additional to visually grading 3D blood flow patterns, we focused on the quantitative analysis of alterations in pulsatile blood flow velocities and wall shear stress known to affect endothelial cell function and thus vessel wall architecture potentially as well [8,9].

Previous investigations with a limited number of patients indicated that blood flow is altered in thoracic aorta aneurysms [11,23,24]. A recently published study [7] investigated blood flow and vessel wall parameters in AAo aneurysms using 4D Flow. In contrast to this study our study analysed all aortic segments (AAo, aortic arch, and DAo and included a larger number of patients. In addition, our study included a comparison of aortic hemodynamics with age matched controls and healthy volunteers.

As demonstrated in previous studies, helix flow in the aorta is a normal physiological phenomenon, moreover it is thought to protect against atherogenesis [18,25]. The present study demonstrates that the incidence of alterations in blood flow patterns such as the presence of vortex and supra-physiologic helical flow was significantly increased in the setting of the dilated ascending aorta. In addition to absolute AAo diameter, the presence and strength of the ascending aortic supra-physiologic-helix and vortex formation in the AAo associated closely with an increase in AAo/DAo diameter ratio. We speculate that in the dilated aorta the expansion results in a altered pressure gradient which can induce secondary flow patterns (i.e. helix and vortex flow) as observed in our patient cohort.

Consistent with the irregular flow patterns observed by visual grading, significant alterations in vessel wall parameters and blood flow velocities were noted. We observed considerable differences among the three cohorts for both pulsatile velocity-time and WSS-time curves. Although differences between patients and age-matched controls were less pronounced, statistically significant alterations were detectable at multiple anatomical locations in the thoracic aorta. Time-to-peak systolic velocities and the extent of retrograde flow were considerably higher in subjects with a dilated aorta, consistent with reduced flow efficiency compared to age-matched controls. With respect to vessel wall parameters, peak systolic WSS and OSI exhibited the largest differences among the different cohorts.

It is well known that reduced shear stress induces vessel constriction, whereas greater shear stress is followed by dilatation through enhanced endothelial nitric oxide synthase activity to maintain mean arterial WSS balance as long as the endothelial function is intact [9,26-28]. Chronically lowered or oscillatory shear stress activates monocytes, and increases platelet activation, oxidant state, apoptosis and cellular turnover. These changes are followed by endothelial cell loss and desquamation, lipid accumulation, platelet aggregates; fibrin plugs are diminished in actin stress fibers and proliferation of smooth muscle cells [9,29]. Such changes in vessel wall architecture can reduce aortic distensibility [6]. These changes of the vessel wall can lead on the one hand to the initiation and progression of atherosclerosis. AAo aneurysms are associated with increased level of matrix metalloproteinase (MMP) which protects from atherosclerosis [1]. These observations and the pronounced supra-physiologic-helix flow could explain that atherosclerosis is not a feature of AAo aneurysms, although WSS is lowered. On the other hand, there is evidence that cerebral aneurysms in humans develop in regions of low WSS [8,30], whereas high WSS may be responsible for initiating aneurysm formation [30]. The findings in our study indicate that the situation for aortic aneurysms may be similar. One explanation could be that the reduced aortic distensibility in AAo aneurysms restricts vasoconstriction of the vessel [6]. However, longitudinal studies correlating changes in WSS with aneurysm progression are needed to support this hypothesis.

High OSI has effects similar to low WSS concerning atherogenesis and vessel wall architecture [9], and is also suspected of playing a role in aneurysm initiation and development.

The decrease in systolic WSS and increase in OSI in patients with dilated AAo observed in this study is well in line with previous studies identifying low WSS and high OSI as critical wall parameters indicative of vascular remodeling and aneurysm development [8,9,29]. Moreover, the observed blood flow patterns in patients are associated with asymmetric changes in regional peak systolic WSS in the AAo and proximal arch. Notably, significant reductions in regional peak systolic WSS were centered along the right outer aortic wall. Interestingly, the identified location of peak systolic WSS matches the predominant location of intimal tears in aortic dissection [31]. Our findings clearly indicate that peak systolic WSS alterations are inconsistently distributed over the vessel lumen circumference. It is conceivable, that regions with low WSS are predisposed to local vessel wall weakening. Importantly, in patients with dilated AAo vessel wall parameters were not only altered at the level of aortic dilation but also in the vessel segments distal to the dilation, implying that an enlarged AAo influences vessel wall parameters in still-healthy segments.

A limitation of our study is the fact that 3D blood flow visualization with particle traces may be diminished when passing through regions of complex flow or areas with high velocity noise [32,33]. We did not test different particle seeding schemes. Nevertheless, we believe that emitting particle traces during systole (i.e. high velocity to noise ratio), which were used for helix grading, provide a reliable tool for identifying bulk flow characteristics such as increased helix flow using our grading scheme. Additional studies are needed to systematically investigate the influence of seeding patterns, particle density and velocity noise on the number, configuration and lengths of particle traces.

A further draw back ouf our study is related to the data analysis strategy for the characterization of aortic hemodynamics. 3D flow visualization and semi-quantitative grading is more sensitive to observer variability and less robust compared to automated and fully quantitative methods [25,34]. Fully quantitative ranking of flow topologies was not available at the time of the analysis of our data and we think that such tools may have the potential to improve flow pattern detection and analysis robustness. Future studies will benefit from more sophisticated analysis as the tools become more mainstream.

Additional limitations of this study are the plane-wise, and not genuine 3D quantification, and the limited spatial and temporal resolution of the MR technique. The aortic root was excluded in this study due to the high movement of the aortic root and proximal aortic bulb over the cardiac cycle. As the analysis planes are in a static position, quantification of flow and particularly WSS was thus deemed unreliable. Software and hardware improvements are necessary that allow quantifications of derived wall parameters along the entire aortic lumen and not just of a given analysis plane in a specific vessel segment. Limited spatial and temporal resolution can lead to underestimation of the genuine WSS magnitude, as shown previously in studies comparing CMR measurements and WSS derived from computational fluid dynamics (CFD) [35]. Nevertheless, the MR-derived WSS in this study is concordant with results of other studies [36]. Furthermore, WSS was not only assessed in patients with dilated AAo but also in age-matches controls, otherwise subject to the same technical bias. Finally, patients were retrospectively selected from a larger cohort and not enrolled prospectively or consecutively, thus subject to selection bias. Follow-up studies of patients and age-matched controls are warranted to evaluate the predictive value of the flow and WSS alterations we have detected regarding further dilatation and/or aneurysm development. The focus of such follow-up studies should be process of aortic dilatation in the patient group so as to identify any correlations between aneurysm development and blood flow and vessel wall parameters alterations.

## Conclusions

This study shows that there is a significant relationship between the ascending aorta diameter as well as the ratio of the ascending and descending aorta diameter and the incidence and strength of irregular blood flow. These findings indicate that peak systolic WSS may be a risk factor for aortic dilation in regions with low WSS, however longitudinal studies correlating changes in WSS with aneurysm progression are needed to support this hypothesis. In addition, vessel wall parameters in patients with dilated AAo were altered at the dilation level as well as in the vessel segments distal to the dilation, highlighting that an enlarged AAo influences vessel wall parameters in adjacent normal segments.

## Competing interests

The authors declare that they have no competing interests.

## Acknowledgement

Grant support by the National Heart, Lung, And Blood Institute of the National Institutes of Health under Award Number R01HL115828 and the Northwestern Memorial Foundation Dixon Translational Research Grants Initiative (MM).

## Authors' contributions

JB: flow visualization, flow quantification, figures, manuscript, flow grading; PB: manuscript, statistics, data evaluation; ZS: flow grading, manuscript; AB: flow quantification, flow data analysis, figures, manuscript; MR: flow visualization, flow quantification, manuscript; JG: flow visualization; AF: study design, manuscript revision; ML: manuscript revision, supervision; MM: study design, flow quantification, flow data analysis, statistics, figures, manuscript revision, supervision. All authors read and approved the final manuscript.
